# Proprioceptive and tactile processing in individuals with Friedreich ataxia: an fMRI study

**DOI:** 10.3389/fneur.2023.1224345

**Published:** 2023-09-22

**Authors:** Virginie Destrebecq, Antonin Rovai, Nicola Trotta, Camille Comet, Gilles Naeije

**Affiliations:** ^1^Laboratoire de Neuroanatomie et de Neuroimagerie translationnelles (LN^2^T), UNI – ULB Neuroscience Institute, Université libre de Bruxelles (ULB), Brussels, Belgium; ^2^Department of Neurology, CUB Hôpital Erasme, Université libre de Bruxelles (ULB), Brussels, Belgium

**Keywords:** Friedreich ataxia (FA), fMRI, proprioception, tactile stimulation, somatosensory system

## Abstract

**Objective:**

Friedreich ataxia (FA) neuropathology affects dorsal root ganglia, posterior columns in the spinal cord, the spinocerebellar tracts, and cerebellar dentate nuclei. The impact of the somatosensory system on ataxic symptoms remains debated. This study aims to better evaluate the contribution of somatosensory processing to ataxia clinical severity by simultaneously investigating passive movement and tactile pneumatic stimulation in individuals with FA.

**Methods:**

Twenty patients with FA and 20 healthy participants were included. All subjects underwent two 6 min block-design functional magnetic resonance imaging (fMRI) paradigms consisting of twelve 30 s alternating blocks (10 brain volumes per block, 120 brain volumes per paradigm) of a tactile oddball paradigm and a passive movement paradigm. Spearman rank correlation tests were used for correlations between BOLD levels and ataxia severity.

**Results:**

The passive movement paradigm led to the lower activation of primary (cSI) and secondary somatosensory cortices (cSII) in FA compared with healthy subjects (respectively 1.1 ± 0.78 vs. 0.61 ± 1.02, *p* = 0.04, and 0.69 ± 0.5 vs. 0.3 ± 0.41, *p* = 0.005). In the tactile paradigm, there was no significant difference between cSI and cSII activation levels in healthy controls and FA (respectively 0.88 ± 0.73 vs. 1.14 ± 0.99, *p* = 0.33, and 0.54 ± 0.37 vs. 0.55 ± 0.54, *p* = 0.93). Correlation analysis showed a significant correlation between cSI activation levels in the tactile paradigm and the clinical severity (R = 0.481, *p* = 0.032).

**Interpretation:**

Our study captured the difference between tactile and proprioceptive impairments in FA using somatosensory fMRI paradigms. The lack of correlation between the proprioceptive paradigm and ataxia clinical parameters supports a low contribution of afferent ataxia to FA clinical severity.

## Introduction

Friedreich ataxia (FA) is the most frequent recessive cerebellar ataxia and affects approximately 1 in 50,000 Caucasians. FA is mainly caused by expanded GAA triplet repeats in the first intron of the frataxin (FXN) gene (GAA1). The GAA1 triplet expansion size correlates with age at onset and disease severity (1). Clinically, FA symptoms generally begin around puberty with progressive gait and limb ataxia that requires, after 10 to 15 years of disease, a wheelchair and help in daily life activities (2).

FA neuropathology affects dorsal root ganglia (DRG), spinocerebellar tracts, and posterior columns in the spinal cord, followed by progressive atrophy of the cerebellar dentate nuclei and dentato-thalamo-cortical tracts (3). The ataxic symptoms of patients pertain to proprioceptive deficit and cerebellar dysfunction (4). In his seminal description, Nikolaus Friedreich identified that the disease affects the dorsal columns over the entire length of the spinal cord and, to a lesser degree, the anterolateral columns (5). In DRG, nerve size is significantly reduced, with a distribution skewed toward a smaller size but with normal axonal count per area (6). Dorsal roots are thin and lack large axons and thick myelinated fibers (3). There are inconsistencies between studies that found that the DRG and spinal abnormalities occur early in development and remain stable over time (7–9), arguing for hypoplasia and studies that show that the DRGs of patients with long disease duration still display signs of active inflammation, supporting a continuing degenerative process (10). Proprioception and tactile perception rely on Aβ myelinated fibers that convey information to the hemispheric and cerebellar cortices through the lemniscal pathway in the dorsal column of the spinal cord and spinocerebellar tracts that are located laterally in the spinal cord. The peripheral sensory nerves that transduce somatosensory stimulations are also affected in FA, with impoverished cutaneous innervation of both myelinated and unmyelinated fibers, as well as thinning of myelinated fibers (6, 11–13). The progression of peripheral nerve pathology is, similar to DRG and spinal posterior column alteration, debated between studies showing stable anomalies over 6 years of follow-up (14) and studies indicating that the density of myelinated fibers is inversely proportional to the patient’s age at the time of biopsy (12). Evidence for progressive impairment of tactile perception also came from a study that systematically quantified epidermal nerve fiber density (ENFD), sensory thresholds (QST), and Meissner corpuscle (MC) density (15). Patients with FA showed lower EFND, lower MC density, and higher QST. Those anomalies significantly correlated with disease duration and clinical severity, arguing for progressive alteration (15).

How all those findings translate clinically is not obvious. Clinical series of individuals with FA describe only discrete impairment of light touch and pain perception, especially in the upper limbs (16, 17). For instance, the FA patients of Harding’s cohort showed a higher threshold for light touch detection in only 3.5% of patients compared with altered joint perception in 23% of patients (16). Functional MRI (fMRI) analysis of brain somatosensory area activation could improve understanding of the apparent difference between proprioceptive and tactile impairment and their contribution to FA ataxia severity. Indeed, fMRI activity in cortical somatosensory areas correlates with symptom severity in disorders affecting peripheral nerves and/or the spinal cord (18–20). In FA, even if no fMRI studies investigated somatosensory brain processing, seven studies, reviewed by Vavla et al. (21) used active finger or hand tapping paradigms that showed robust primary and secondary somatosensory activations that probably corresponded to the proprioceptive counterpart of active movements. These studies provided two important pieces of evidence. First, the somatosensory hand and finger cortical representation areas are similar in FA and controls (21). Second, a relationship between somatosensory fMRI activation and clinical severity was identified in some (22, 23) but not all studies (24, 25). Therefore, fMRI somatosensory paradigms could help characterize the somatosensory impairments in FA. This is an important issue, especially in the hands, which are crucial for maintaining the autonomy of individuals with FA who will become wheelchair bound. Better understanding what component of somatosensory transduction, between proprioception and tactile perception, is progressive, may also help target frataxin-restoring therapies and patient care.

The objective of this study is, thus, to investigate proprioception and tactile perception simultaneously in individuals with FA using a pneumatic tactile stimulation paradigm and a passive movement paradigm in functional MRI and search for a correlation between somatosensory processing and disease severity.

## Subjects and method

### Participants

Twenty patients with genetically proven FA (eleven women, mean age 32.6 ± 15, one left-handed) and 20 healthy participants matched for age (±5 years) and handedness (twelve women, mean age 33 ± 3.9 years) devoid of neurologic or psychiatric disease were included (Table 1).

**Table 1:** Characteristics of the included individuals with FA.

Age (mean ± standard deviation; years)	32.6 ± 15
SARA (mean ± standard deviation/40)	23 ± 9
Age of symptoms onset (mean ± standard deviation)	18 ± 14
Disease duration (median ± standard deviation; years)	15 ± 7.5
GAA1 (mean ± standard deviation)	654 ± 268*

SARA, score on the scale for the assessment and rating of ataxia; GAA1, number of GAA1 triplet expansions on the shortest allele. * one patient had a point mutation.

FA individuals were clinically evaluated using the Scale for the Assessment and Rating of Ataxia (SARA) (26), the day they underwent the fMRI investigations.

All participants contributed to the study after written informed consent and prior approval of the study by the CUB Hôpital Erasme Ethics Committee (Reference EudraCT/CCB: B4062021000483).

### fMRI functional imaging

#### Acquisition

All MRI data acquisitions were performed on a hybrid 3 T SIGNA PET-MRI scanner (GE Healthcare, Milwaukee, Wisconsin, United States) using a 24-channel head and neck coil. Functional data were acquired by using single-shot gradient-echo echo-planar-imaging (GE/EPI) T2* weighted-images (WI), covering the whole brain (time of repetition [TR]/time of echo [TE]/flip angle [α], 3,000 msec/34 msec/90°; field of view, 28 cm; acquired matrix, 96 × 96; slice thickness, 3 mm; in-plane resolution, 2.9 × 2.9 mm; 40 slices). Four dummy scans (total duration, 12 s) were obtained before each session to allow the MR signal to reach a steady state and were subsequently automatically discarded by the scanner. A three-dimensional T1-weighted GE sequence covering the whole brain was acquired in all subjects to co-register the functional data (TR/TE/α: 8.3 msec /3.1 msec/12°; field of view: 24 cm; matrix: 240 × 240; slice thickness: 1 mm; in-plane resolution 1 × 1 mm), as in Lolli et al. (27).

#### fMRI paradigm

All subjects underwent two 6 min block-design fMRI paradigms consisting of twelve 30 s alternating blocks (10 brain volumes per block, 120 brain volumes per paradigm). The tactile oddball paradigm (*oddball*) was adaptated from Naeije et al. (9, 28). Stimuli were applied using a pneumatic stimulator, as described by Wienbruch et al. (29). Standard stimuli were applied to the right index fingertip (stimulated area, 1 cm2; intensity, 3.5 bars; duration, 100 ms, inter-stimuli interval, 500 ms), while deviants consisted of the simultaneous stimulation of the fingertip and middle phalanx of the right index finger. Standard and deviant stimuli were randomly interspersed with a ratio of four standards/one deviant. This oddball paradigm was chosen for its reproducible activation of the somatosensory cortices in both healthy individuals (30, 31) and individuals with FA (9). The passive movement paradigm (*CKC*) used a pneumatic artificial muscle (PAM) stimulator that consists of two embedded plastic cylinders. The inner cylinder slides rhythmically back and forth thanks to the action of a custom-made elastic actuator that generates computer-controlled passive finger movements by shortening with increasing air pressure (0–4 bar). The subject’s index finger is fixed with Velcro straps to the upper plate of the smaller cylinder. The longitudinal shortening of the PAM (approximately 25 mm displacement) due to increasing internal air pressure and subsequent retraction resulted in a flexion of the index finger. Flexion is followed by an extension of the index finger when the PAM internal air pressure returns to normal [for technical details, see Lolli et al. (27)]. The *CKC* paradigm consisted of twelve alternating blocks of passive movement and rest. During the “movement” blocks, the PAM-based stimulator induced repetitive passive flexion-extensions of the right index fingers of the subjects at a fixed rate of 3 Hz (approximately 2.5 mm displacement) (27). This passive movement paradigm was selected as passive movements reflect proprioceptive afferences to the somatosensory cortex (32) and because a similar paradigm in magnetoencephalography led to an altered cortical evoked response in individuals with FA (33).

#### Data analysis

fMRI data preprocessing analyses were performed using SPM12 software (Wellcome Department of Imaging Neuroscience, London, United Kingdom, https://www.fil.ion.ucl.ac.uk/spm/), implemented in Matlab (2017a, Mathworks Inc., Sherbom, MA, United States) using a conventional pipeline of analysis detailed by Lolli et al. (27).

Mean individual framewise displacements were computed for each subject and compared between healthy and FA individuals using a two-sample *t*-test, as described by Lolli et al. (27).

#### First-level analysis

Functional images were preprocessed using slice timing correction, realignment, co-registration to the patients’ corresponding structural images, normalization to the Montreal Neurological Institute (MNI) template, and smoothing [obtained by applying an isotropic Gaussian kernel of 8 mm fullwidth at half maximum (FWHM)]. A high-pass filter was applied to remove signal drifts with a period longer than 128 s (27). Statistical analyses were performed in the general linear model (GLM) framework. For each patient, we constructed one GLM for each condition that included the preprocessed fMRI data in which the experimental conditions (i.e., tactile oddball stimulation vs. rest) were modelled as boxcar functions convolved with the canonical hemodynamic response function. The GLMs also included, as co-variates of no interest, the corresponding six motion parameters obtained from realignment (27, 34, 35). First-level (within patients) statistical T maps were then created to identify significant increases in blood-oxygen-level-dependent (BOLD) signal between stimulation paradigms vs. rest conditions (positive BOLD response, PBR), as well as decreases (negative BOLD responses, NBRs) in the tactile oddball paradigm. NBRs were studied in the tactile paradigms according to evidence that shows unstimulated sensorimotor cortex NBR ipsilateral to unilateral hand movements or somatosensory stimuli (36–38). Statistical T-maps were thresholded voxelwise at *p* < 0.05 (family wise error rate [FWE] corrected for multiple comparisons; extent threshold, *k* = 0).

Then, given our strong *a priori* hypotheses about the activation of primary and secondary somatosensory cortices based on previous fMRI results using similar paradigms (27, 29–31), we used a region of interest (ROI) approach to compare the BOLD responses of somatosensory cortices in both groups and paradigms with identical statistical significance levels to the whole brain analysis. ROI were defined as 5 mm spheres built around MNI coordinates using the MarsBaR toolbox for SPM12. The contralateral and ipsilateral primary somatosensory cortices’ ROI (cSI-ROI and iSI-ROI), as well as contralateral and ipsilateral secondary somatosensory ROI (cSII-ROI and iSII-ROI), were defined in the passive movement paradigm as 5 mm spheres centered around voxels located, respectively, at the cSI and cSII cortex (MNI coordinates [−34, −36, 70] and [−46, −20, 24]) and the iSI and iSII cortex (MNI coordinates [56, −10, 42] and [56, −18, 20]) using MNI coordinates from Lolli et al. (27). For the tactile oddball paradigm, we used 5 mm sphere ROI for cSI and cSII (MNI coordinates [−48, −22, 46] and [−45, −25, 18]) and iSI and iSII (MNI coordinates [48, −22, 43] and [48, −31, 16]) based on Wienbruch et al. (29). These MNI coordinates are consistent with SI and SII cortex in neuroimaging data meta-analyses (39, 40).

Within each defined ROI, contrast estimates for each subject and for each voxel were extracted. Then, a single value for each subject at each ROI was computed by averaging contrast estimates over all the voxels within each ROI. ROI values between FA and healthy individuals in both conditions were compared using a two-sample unpaired *t*-test. *p* < 0.05 was considered significant.

#### Second-level analysis

For group analyses, the contrast images resulting from the tactile oddball and passive movement paradigms in FA and healthy individuals were entered into a second level analysis, using a Random Effects Model (RFX). Non-parametric permutation tests (number of permutations 5,000) were used to assess group-level whole-brain peak activations across sessions for both paradigms in each group (41) using the SnPM toolbox. This process generates pseudo-T statistic maps that correspond to statistical T maps in which variances are smoothed, a procedure that prevents the problem of artefactually high statistic values in regions with both low signal and low noise (42). As for the first-level analyses, the significance level for the resulting statistical maps was initially set at *p* < 0.05 FWE (extent threshold *k* = 0) or at uncorrected *p* < 0.001 if no clusters survived FWE correction. This was carried out to see whether the activated networks were congruent with previous studies.

### Correlation analysis

Spearman rank correlation tests were used to search for possible relationships between the BOLD levels of individuals with FA at the cSI, iSI, cSII, and iSII in both conditions and the size of GAA1 triplet expansion, and the SARA score. Somatosensory cortex activity correlating with GAA1 would argue for a developmental and stable deficit while correlation with the SARA would support a link between somatosensory system function and the progression of ataxic symptoms. Of notice, the patient with a point mutation in the *FXN* gene was excluded from the correlations with GAA1 triplet expansion. Results were considered statistically significant at *p* < 0.05.

## Results

The group average of means of individual framewise displacements was significantly slightly higher in FA individuals (FA, 0.28 ± 0.23 mm vs. 0.12 ± 0.06 mm, *p* = 0.005).

### fMRI analysis

#### Group-level analysis

Both conditions in both groups consistently lead to a significant increase in BOLD signal at the contralateral primary somatosensory cortex and bilateral secondary somatosensory cortices.

Table 2 lists the MNI coordinates derived from the peak voxels and the corresponding functional brain region for *oddball* and *CKC* group analysis in individuals with FA and healthy controls.

**Table 2:** Brain regions showing a significant increase in BOLD signal when contrasting the *oddball* and *CKC* condition versus rest at the group level in individuals with FA and healthy controls.

Anatomical region	MNI coordinates [x, y, z]	Pseudo-T values	*Ke*	*p*
Healthy controls				
cSI	[−58, −20, 46]	6.68	1,608	0.0028^*^
cSII	[−52, −18, 22]	7.31	1,608	0.0028^*^
iSII	[66, −18, 18]	5.20	246	0.0054^*^
CKC-cSI	[−46, −18, 54]	7.99	3,422	0.0018^*^
CKC-cSII	[−53, −28, 20]	6.5	3,422	0.0036^*^
CKC-iSII	[55, −28, 17]	5.66	1,167	0.0044^*^
CKC-lCerebVI	[28, −52, −26]	4.39	184	0.048^*^
Individuals with FA				
cSI	[−54, −18, 52]	7.5	255	0.0002^*^
cSII	[−52, −20, 20]	5.28	18	0.0224^*^
iSII	[50, −24, 22]	5.26	8	0.0023^*^
CKC-cSII	[−50, −22, 22]	3.49	125	0.0002^•^

^*^_few-corr._^•^*uncorr*. *Ke*, size of the clusters in the number of voxels.

**Figure 1:**
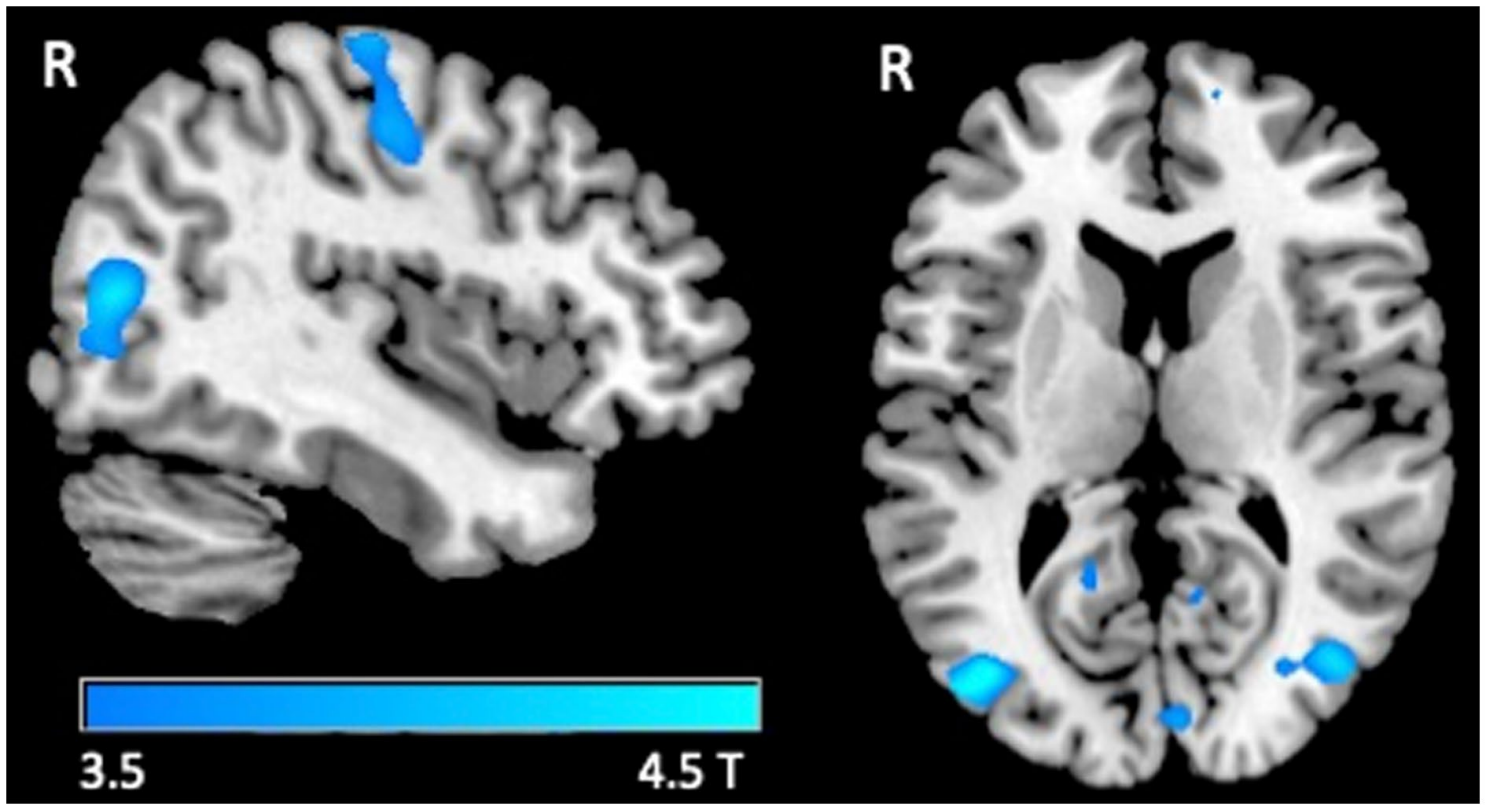
fMRI response. Group-level T maps of the de-activations induced by the oddball tactile paradigms in healthy individuals.

A significant decrease in BOLD signal was only observed at the right sensorimotor cortex and bilateral visual areas in healthy subjects in the tactile oddball condition (Figure 1; Table 3).

**Table 3:** Brain regions showing a significant decrease in BOLD signal when contrasting the *oddball* versus the rest at the group level in healthy controls.

Anatomical region	MNI coordinates [x, y, z]	*T* values	*Ke*	*p_uncorrected_*
Healthy controls				
Right precentral	[−58, −20, 46]	4.52	54	<0.0001
Right occipital	[−42, −78, 12]	4.15	307	<0.0001
Right calcarine	[−4, −88, 20]	4.6	227	<0.0001
Left calcarine	[6, −88, 14]	4.26	227	<0.0001
Left occipital	[40, −72, 12]	4.82	135	<0.0001

*Ke*, size of the clusters in the number of voxels.

#### Subject-level analysis

For the *tactile oddball* condition, there was no significant difference between the cSI-ROI, cSII-ROI, and iSII-ROI mean beta estimates in individuals with FA and healthy controls (respectively 0.87 ± 0.57 vs. 0.77 ± 0.52, *p* = 0.6; 0.69 ± 0.57 vs. 0.63 ± 0.39, *p* = 0.64, and 0.36 ± 0.28 vs. 0.37 ± 0.36, *p* = 0.87).

**Figure 2:**
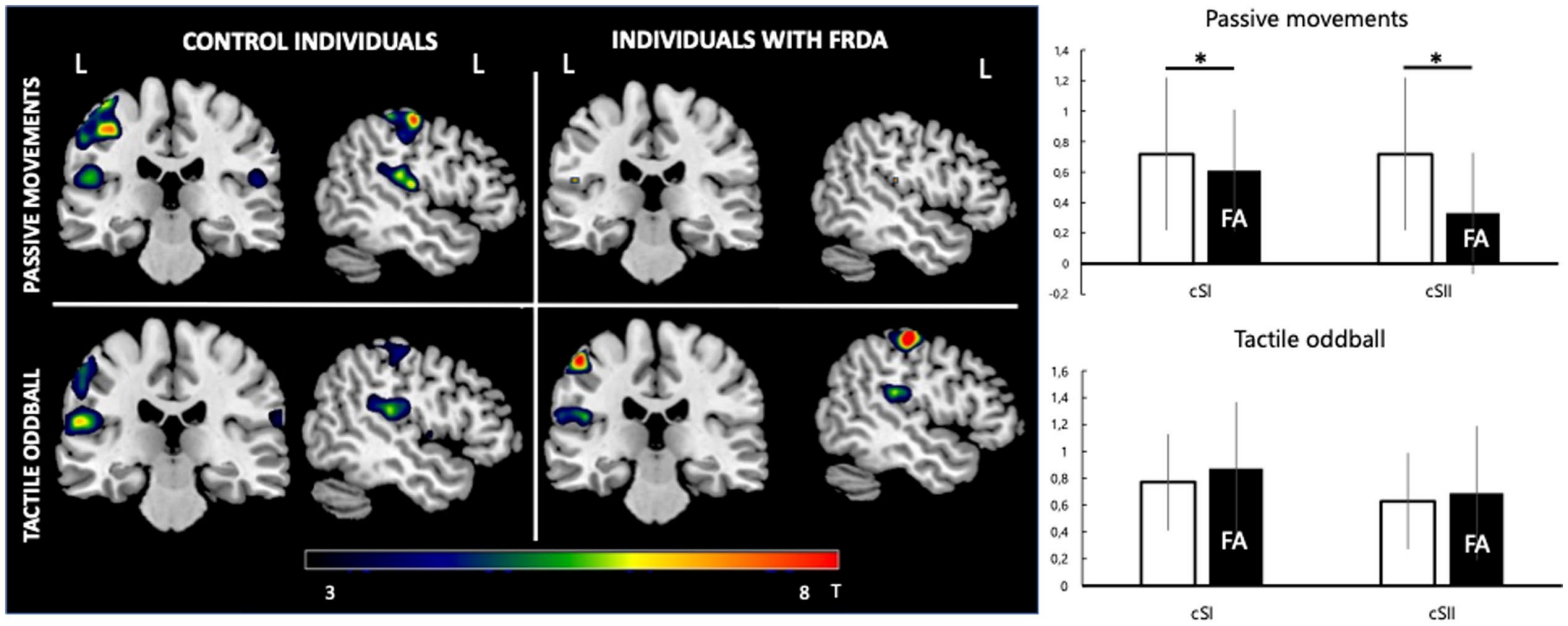
fMRI response. Left: group-level pseudo-T maps of the activations induced by the passive movement and oddball tactile paradigms in healthy individuals (left column) and FA patients (right column). Right: fMRI response at the left primary somatosensory cortex (cSI) and left secondary somatosensory cortex (cSII) ROI in the passive movement condition and oddball tactile condition (bottom). Y-axis values correspond to mean beta estimates. Mean FA patient values are represented in the black histogram. *Significant difference between healthy and FA individuals (*p* < 0.05).

For the *passive movement* condition, there was a significant difference for cSI-ROI and cSII-ROI BOLD levels in individuals with FA and healthy controls (respectively 0.61 ± 0.81 vs. 0.72 ± 0.79, *p* = 0.002 and 0.34 ± 0.42 vs. 0.72 ± 0.49, *p* = 0.013). There was significant activity at iSI (0.38 ± 0.48) and iSII (0.35 ± 0.28) ROI only in healthy controls (Figure 2).

**Figure 3:**
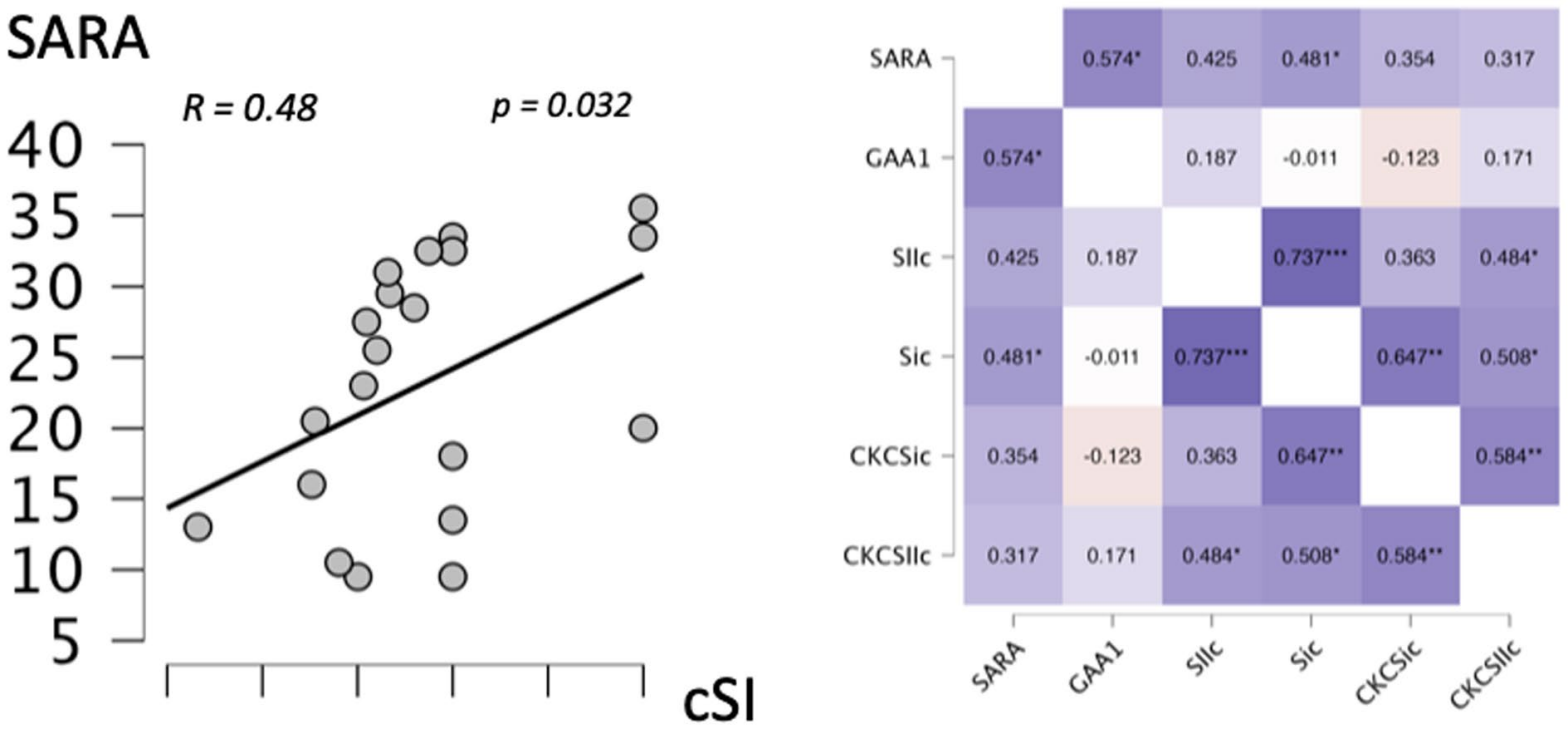
Correlation. Left: graphical representation of the correlation between the left primary somatosensory cortex (cSI) in the tactile paradigm and the SARA score. Right: heatmap of the correlations between left primary somatosensory cortex (SI) and left secondary somatosensory cortex (SII) ROI in the oddball condition, left primary somatosensory cortex (CKC_SI), and left secondary somatosensory cortex (CKC_SII) ROI in the oddball condition, the genotype (GAA1 expansion size), and the SARA score. *Significant correlation (*p* < 0.05); **Significant correlation (*p* < 0.01).

Correlation analysis showed a significant correlation between cSI-ROI BOLD levels in *the tactile oddball condition* and SARA (R = 0.481, *p* = 0.032) (Figure 3 displays the correlation analysis heatmap and the graphical representation of the significant correlations).

## Discussion

The main findings from this study are that only the proprioceptive paradigm led to a lower activation of somatosensory cortices in individuals with FA compared to healthy subjects while the tactile oddball led to a similar activation of somatosensory cortices in both groups, reflecting the clinical difference observed for proprioceptive and tactile impairment in FA.

Despite the relatively modest sample size of our study, we believe that our results are likely to be generally valid. Our population of individuals with FA closely shares characteristics in terms of age, age of onset, GAA1 expansion size, and SARA score with the large natural history cohorts of individuals with FA (1, 4, 43).

### Somatosensory network activation

Passive movements have been shown to reflect proprioceptive afferences to somatosensory cortices across different functional neuroimaging techniques from positron emission tomography (PET) (44) to fMRI (27) and magnetoencephalography (MEG) (32). Passive movements robustly activate cSI and cSII across all functional imaging modalities (44–46). Our results for healthy individuals reproduce the findings usually reported for fMRI for passive movements, with the activation of the cSI, bilateral SII, and ipsilateral cerebellum (27, 45). In individuals with FA, to the best of our knowledge, no study has assessed somatosensory cortices activation in fMRI passive movement paradigms. However, several studies have evaluated the BOLD levels of somatosensory cortices for active finger tapping movement in FA patients and described lower cSI (25) and lower cSII BOLD levels (22) than in healthy subjects. Somatosensory cortices activation in our passive finger movement paradigm can be compared with the results obtained with active finger flexion/extension movements. Several studies that contrasted active and passive movements in PET (47), fMRI (27, 48), and MEG (49) described similar activity at the somatosensory cortices in both conditions, suggesting that the somatosensory cortices activity in passive and active movement paradigms reflects the proprioceptive component of movement.

### Passive movement and proprioception in FA

The lower activation of the cSI for passive movements in individuals with FA parallels the findings of a previous MEG study that found significantly lower cSI evoked responses for passive and active movements but similar neural generators than in controls (33). Therefore, the lower BOLD level activity at the cSI and cSII, observed in individuals with FA, likely reflects the proprioceptive impairment in FA individuals. This lower activation at somatosensory cortices in FA could also relate to more variable cortical representation of the finger at the surface of the somatosensory cortex due to altered sensory afferences. However, fMRI studies in finger or hand active movement showed similar somatosensory area activation in FA to controls which argues against this interpretation (21).Proprioceptive information also travels directly to the cerebellum through the spinocerebellar tracts and leads to posterior cerebellar activation in fMRI passive movement paradigms (27), as reflected in our healthy subjects. The lack of cerebellar activation for passive movement, in our individuals with FA, is probably the consequence of the atrophy of the spinocerebellar tracts observed with FA neuropathology (3) and may further contribute to the proprioceptive counterpart of FA ataxic symptoms. It must be kept in mind that passive movements are not exclusively associated with the proprioceptive aspects of the movements as they also stimulate the cutaneous receptors at the surface of the skin. Therefore, the lower activation at somatosensory cortices observed in FA individuals could also be influenced by an impairment of cutaneous nerves fibers that adds to the proprioceptive information transmitted to somatosensory areas. However, the fact that in the tactile oddball, no differences were observed at somatosensory cortices between FA and healthy individuals suggests that skin cutaneous receptors may have a limited influence compared with proprioceptive feedback in our passive movement paradigm. Finally, FA individuals displayed slightly more framewise displacements than healthy subjects, which may have impacted their activation values. Yet, as no differences in activations were found in the other condition between the two groups, this discrete increase of framewise displacement in FA probably had negligible effects.

### Tactile stimulation and somatosensory cortices responses in FA

The tactile oddball paradigm led to the expected activation in the cSI and cSII in individuals with FA and healthy controls (29, 50, 51). Strikingly, there were no differences between individuals with FA and healthy controls either in the cSI or cSII BOLD levels for the tactile pneumatic stimulation. Similar BOLD levels at the cSII for tactile stimulation were expected as cSII responses do not depend on stimulation intensity but on the more complex features of tactile stimulation and attentional status (31, 52). The lack of difference at the cSI either supports the fact that tactile processing at sensory cortices is less affected than the proprioceptive processing or that cSI BOLD levels fail to capture a more discrete alteration, as a MEG study using a similar tactile oddball paradigm found lower evoked responses at the cSI in FA patients (9). Comparable levels of activation at somatosensory cortices, in FA patients and controls, corroborates the clinical observation that individuals with FA seem to adequately feel the stimulation when they are touched or manipulate objects and rarely display significant anomalies on routine clinical tact evaluation (16, 17). However, our results must be balanced with the fact that individuals with FA have lower epidermal nerve fiber densities, lower Meissner corpuscles densities, and impaired tactile QST. Such a normal response at somatosensory cortices in individuals with FA could relate to a combination of factors. First, cSI BOLD levels are a function of the stimulus intensity (53). The pneumatic stimulation used in this study had an intensity that was above the higher tactile QST thresholds described for FA patients and may partly explain why similar cSI BOLD levels were found in both groups. Second, fMRI may lack sensitivity for subtle tactile impairment. The alteration of the tactile QST thresholds described in FA is significant but quantitatively modest (approximately 0.2 gr compared with approximately 0.1 gr) with respect to bending the von Frey filament in healthy individuals. Therefore, such a difference is likely to escape routine clinical testing in a similar way as it escapes fMRI detection (15, 54). Interestingly, the FA range of tactile impairment corresponds to the ranges observed in older healthy individuals in which the increase in tactile QST and the reduction in Meissner corpuscle density occur in a similar proportion (55, 56). Additionally, healthy older individuals and individuals with FA have in common more obvious proprioceptive abnormalities than tactile impairments (57) and the fact that their tactile impairments are not associated with different responses at the cSI for tactile stimulation (58). The tactile impairment of FA patients may not be clinically relevant at our mean group age of 32 years old, but continuous degradation of peripheral skin tactile receptors due to age in patients with a lower receptor “reserve” could lead to clinically significant impairment as they become older. The stable impairment of the afferent tracts of the spinal cord dorsal column, reported by structural longitudinal spinal cord imaging (59), is now challenged by the fact that microstructural dorsal column diffusion tensor imaging describes a correlation between clinical severity and dorsal column diffusion parameters (60) that is in line with the correlations between MC density, ENFD, and clinical severity. This suggests that tactile impairment, even if modest, may be progressive (15). In that context, longitudinal fMRI with the tactile paradigm coupled to QST studies could help disentangle the potential lack of progression from fMRI lack of sensitivity. Finally, another possible explanation for the relatively preserved tactile perception in FA and cSI BOLD levels could relate to the other epidermal tactile receptors. MC are rapidly adapting low-threshold mechanoreceptors that briefly respond at the beginning and end of the stimulation. Merkel discs, on the other hand, are slowly adapting low-threshold mechanoreceptors that respond during the whole touch-pressure experience (61). Merkel discs in the finger skin have a five to tenfold density compared with MC and may compensate for the eventual loss of tactile information due to lower MC density (62). To date, the potential alteration of Merkel disc density or functionality and their role in FA tactile processing have not been studied and warrants further investigation. Finally, normal positive bold responses (PBRs) at the cSI may not reflect normal physiological responses to tactile stimulation. Indeed, in healthy individuals, PBRs at the cSI are associated with negative bold responses (NBRs) at the ipsilateral sensorymotor cortex (iSMI) that reflect physiological interhemispheric inhibition (IHI) integrity through the corpus callosum (36, 38). Such NBRs at the iSMI, present in our healthy individuals group, were lacking in individuals with FA, which suggests that cSI PBRs may be falsely normal in part because of a loss of inhibition from the iSMI. This loss of interhemispheric inhibition in FA could be due to the functional impact of the microstructural anomalies observed in FA individuals’ corpus callosum (63). Alternatively, IHI impairment could also implicate cerebello-cortical disconnectivity as other degenerative diseases that display IHI impairment such as Alzheimer Disease (64) and amyotrophic lateral sclerosis (65) also present with cerebello-cortical alterations (66, 67).

### Correlation between BOLD signal and clinical parameters in FA

The activation levels at the cSI positively correlated with clinical severity in the oddball paradigm and can also be interpreted under the cerebellar inhibition framework. In healthy individuals, cerebello-cortical loops have an inhibitory action on the neocortex. This cerebellar inhibition is altered in cerebellar diseases and leads to higher cortical excitability (68–70). The fact that cSI responses were higher in patients with higher clinical disability suggests a relationship between somatosensory cortex activation and the disconnection from the cerebellum due to the dentato-thalamo-cortical tract impairment in FA, the dysfunction of which correlates clinical severity (63). The lack of significant cerebellar activations in our FA cohort prevented us studying connectivity through psychophysiological interactions and, thus, this interpretation is only hypothetical and should be confirmed in dedicated connectivity studies. However, a recent unilateral cerebellar transcranial direct current stimulation study described a decrease in oxyhemoglobin concentrations in the contralateral sensory-motor cortex and a significant increase in oxyhemoglobin concentrations in the ipsilateral sensory-motor cortex, mirroring the IHI phenomenon (71), which suggests that IHI may also involve the cerebellum.

### Summary

Our study captured the difference between tactile and proprioceptive impairments in individuals with FA using somatosensory fMRI paradigms. The lack of correlation between the proprioceptive paradigm and clinical parameters supports a low contribution of afferent ataxia to clinical severity in FA. The observed pattern of proprioceptive alterations that are more prominent than tactile alterations is similar to age-related somatosensory impairment. In that context, those somatosensory fMRI paradigms could prove to be important tools in longitudinal studies assessing how age, FA, and disease duration could potentiate each other in making tactile perception loss clinically relevant.

## Data availability statement

The raw data supporting the conclusions of this article will be made available by the authors, without undue reservation.

## Ethics statement

The studies involving humans were approved by Comité d’éthique Hospitalo-facultaire Erasme-ULB. The studies were conducted in accordance with the local legislation and institutional requirements. Written informed consent for participation in this study was provided by the participants.

## Author contributions

VD conducted the experiments, analyzed the data, and wrote the manuscript for intellectual content. AR analyzed the data and drafted the manuscript for intellectual content. NT and CC conducted the experiments and drafted the manuscript for intellectual content. GN design and conceptualized study, conducted the experiments, analyzed the data and wrote the manuscript. All authors contributed to the article and approved the submitted version.
